# Somatic hypermutation shapes the viral escape profile of SARS-CoV-2 neutralising antibodies

**DOI:** 10.1016/j.ebiom.2025.105770

**Published:** 2025-05-21

**Authors:** Matthias Bruhn, Maureen Obara, Mariana Gonzalez-Hernandez, Wencke Reineking, Abdus Salam, Monica Mirolo, Imke Hinrichs, AhmedElmontaser Mergani, Yannic Bartsch, Axel Schambach, Gert Zimmer, Wolfgang Baumgärtner, Albert D.M.E. Osterhaus, Ulrich Kalinke

**Affiliations:** a Institute for Experimental Infection Research, TWINCORE, Centre for Experimental and Clinical Infection Research, a joint venture between the Helmholtz Centre for Infection Research and the Hannover Medical School, Hannover, Germany; b Research Center for Emerging Infections and Zoonoses, University of Veterinary Medicine Hannover, Foundation, Hannover, Germany; c Department of Pathology, University of Veterinary Medicine Hannover, Hannover, Germany; d Junior Research Group Anti-viral Antibody-Omics, TWINCORE, Centre for Experimental and Clinical Infection Research, a joint venture between the Helmholtz Centre for Infection Research and the Hannover Medical School, Hannover, Germany; e Department of Biochemistry, University of Veterinary Medicine Hannover, Hannover, Germany; f Institute of Experimental Hematology, Hannover Medical School, Hannover, Germany; g Division of Hematology/Oncology, Boston Children's Hospital, Harvard Medical School, Boston, MA, USA; h German Center for Infection Research (DZIF), Partner Site Hannover–Braunschweig, Hannover, Germany; i Institute of Virology and Immunology (IVI), Mittelhäusern and Bern, Bern, Switzerland; j Department of Infectious Diseases and Pathobiology, Vetsuisse Faculty, University of Bern, Bern, Switzerland; k Cluster of Excellence RESIST (EXC 2155), Hannover Medical School, Hannover, Germany

**Keywords:** Viral escape, SARS-CoV-2, Monoclonal antibodies, Somatic hypermutation, Binding affinity

## Abstract

**Background:**

Since the onset of the COVID-19 pandemic, SARS-CoV-2 neutralising monoclonal antibodies (mAbs) are being developed for clinical use. With the appearance of new virus variants, most mAbs lost their virus-neutralising activity, highlighting the complexity of mAb development under conditions of continuous SARS-CoV-2 evolution.

**Methods:**

Hamsters were treated with SARS-CoV-2 neutralising mAbs and then challenged with SARS-CoV-2. Recombinant VSV expressing the spike protein of SARS-CoV-2 was utilised in an *in vitro* system to select for antibody escape variants. Surface plasmon resonance measurements were performed to characterise the binding affinity and epitope of various mAbs. Fc-mediated effector functions of neutralising and non-neutralising mAb combinations were determined via multiple *in vitro* assays.

**Findings:**

Few of the mAb treated and infected hamsters experienced breakthrough infections, which derived from mutated virus that emerged *in vivo*. We developed an *in vitro* antibody escape assay that recapitulated the *in vivo* situation and we found that somatic hypermutations (SHM) affected the profile of viral escape hotspots that mAbs selected for. Pairwise combination of mAbs binding non-overlapping epitopes suppressed the emergence of viral mutants. The formulation with a third, non-neutralising mAb enhanced the Fc-mediated effector functions of the mAb treatment in an additive manner.

**Interpretation:**

We conclude that treatment with single mAbs rapidly leads to the formation of novel virus variants. An important function of SHM is to suppress the emergence of viral antibody escape variants. Our data suggest that the anticipatory B cell memory can be harnessed to design combinations of SARS-CoV-2 neutralising mAbs that have a reduced risk to induce viral escape.

**Funding:**

This study was supported by public funding from the 10.13039/501100001659German Research Foundation (DFG), the 10.13039/501100002347Federal Ministry of Education and Research (BMBF), the COVID-19-Research Network of the State of Lower Saxony (COFONI), the 10.13039/100009139German Centre for Infection Research (DZIF), and the 10.13039/501100001656Helmholtz Association of German Research Centres.

**Research in context**

**Evidence before this study**

Fully human monoclonal antibodies (mAbs) are important assets in the arsenal against upcoming viral epidemics as well as pandemics. However, viral mutants can arise that are no longer neutralised by many mAbs. Another challenge is that the clinical application of mAbs itself can drive outgrowth of novel virus variants in patients, a scenario that would further accelerate the appearance and spread of neutralisation-resistant virus strains in the population.

**Added value of this study**

Here, we found in the hamster model that upon treatment with SARS-CoV-2 neutralising mAbs and subsequent SARS-CoV-2 infection, a selective pressure is induced that promotes the emergence of viral antibody escape variants. Multiple strategies were explored to avoid such a scenario. We focused on the analysis of somatic hypermutations within the variable regions of antibodies to reinforce the “Achilles heel” of mAbs, which is exploited by the virus to escape from neutralisation.

**Implications of all the available evidence**

So far, the clinical development of mAbs for the prevention and treatment of COVID-19 largely failed because new virus variants appeared that were no longer neutralised by the mAbs. Therefore, future mAb candidates should be characterised in greater detail for their anticipatory propensity to neutralise viral escape variants. Formulation of synergistic mAb cocktails consisting of several neutralising and non-neutralising mAbs may be advantageous to avoid their loss of activity against evolving viruses.

## Introduction

Due to their high discovery and development speed, monoclonal antibodies (mAbs) were among the first antiviral remedies against SARS-CoV-2 that underwent clinical testing during the COVID-19 pandemic.^1^ Fully human mAbs for clinical application can be generated by different approaches, including phage display, immunisation of humanised mice, and cloning of the variable regions of immunoglobulins that are expressed by B cells from convalescent and/or vaccinated individuals.^2^ In the case of SARS-CoV-2, mAbs were developed by various groups based on their ability to prevent the virus from interacting with the host receptor ACE2, thus leading to neutralisation of the virus.^3^ Although potent neutralising antibody responses were elicited on the population level as a result of recovery from COVID-19 and the rollout of large vaccination campaigns, virus variants of concern (VOC) evolved that are highly infectious and acquired the ability to escape from serum antibody responses as well as from most (if not all) mAbs that were analysed in clinical trials.^3^

During convalescence, SARS-CoV-2-specific B cells undergo affinity maturation to improve the binding and neutralisation strength of their antibodies.^4^ As a result, the humoral immune response is enhanced when convalescent individuals are vaccinated^5^ or when they develop breakthrough infections,^6^ with the restriction that the time between the initial and the subsequent challenge was long enough to allow affinity maturation.

We recently described a set of novel mAbs that were developed from blood donations of COVID-19 convalescent individuals early during the pandemic.^7^ These mAbs potently neutralised SARS-CoV-2, but were susceptible to viral escape from VOC such as Alpha, Beta, Gamma, Delta, and Omicron. Interestingly, when we analysed the effect of somatic hypermutation (SHM), germline versions of the mAbs with reverted SHM were more susceptible to escape by VOC than the respective hypermutated counterparts. This observation is in accordance with other reports^8^^,^^9^ and indicates that VOC predominantly escaped from non-matured plasma antibodies, and were still neutralised by antibodies that underwent “anticipatory” maturation.^7^ Furthermore, we discovered that the formation of an “anticipatory” B-cell memory is likely based on the stochastic diversification of immunodominant clonotypes, which were made up by prevalently used gene segments such as VH3-53.^10^

Here, we follow up on these observations by characterising the preventive potential of three mAbs in an *in vivo* hamster challenge experiment. The affinity of the mAbs, which showed different levels of SHM, was determined by using highly sensitive and precise surface plasmon resonance (SPR) measurements against several VOC. Furthermore, we developed a fast readout technology to identify the “Achilles heel” of different neutralising mAbs by adopting an *in vitro* virus escape assay and carrying out investigations towards how affinity maturation shapes the propensity of SARS-CoV-2 to escape from antibodies at certain immune evasion hotspots in the spike protein. Based on our findings, we hypothesise that the risk to select for viral antibody escape variants can be minimised by combining three mAbs in a cocktail that interact with the receptor binding domain (RBD) simultaneously from three different angles. In such combinations, optimized Fc-mediated effector functions can be advantageous.

## Methods

### Monoclonal antibody sequences

The mAbs that we used in this study were described earlier in detail.^7^^,^^10^ In brief, MB025_A07K and MB025_A09L were isolated from a SARS-CoV-2 convalescent individual early during the pandemic before the occurrence of VOC.^7^ MB027_D06L and 39 other related mAbs (MB043_CL00–CL39) are part of an expanded B cell clonotype, which was observed previously.^10^ In brief, the clonotype was detected by 10X Genomics scRNA-seq analysis with the Next GEM Single Cell 5' Reagent Kit v2 (Protocol CG000330 Rev A). The fastq files were processed using the Cell Ranger v7.1.0 multi command on the human V(D)J reference GRCh38-alts-ensembl-7.1.0. The V(D)J data was processed using the Immcantation suite (v4.4.0). The nearest-neighbour distance distribution method in SHazaM (1.1.2.999) was used for threshold calculation and clones were called via a hierarchical clustering approach (distance-based method) using SCOPer (v1.2.1.999). Lineage trees were constructed and plotted using the Dowser package (v1.1.1) and data were visualised using ggplot2 (v3.4.0) (for more details please refer to Bruhn *et al*., 2024^10^).

### Recombinant expression of monoclonal antibodies

Plasmids that encode for the human heavy IgG1 and the human light chain of the respective mAbs were transfected at a concentration of 0.8 μg/ml into ExpiCHO cells using the ExpiFectamine CHO Transfection Kit following the manufacturer's protocol (Thermo Fisher A29129). For the SPR experiments, 50 ml culture supernatants were produced in 250 ml Erlenmeyer shaker flasks. For the virus escape assays, the mAbs were expressed in 6-well plates in 3 ml culture supernatant per well. For the *in vivo* analysis, 100 ml cultures were grown in 500 ml Erlenmeyer shaker flasks. After 10 days, the cultures were harvested by centrifugation, supernatant was filtered using 0.2 μm syringe filters and stored at −20 °C until further use.

### mAb purification and quality control

For SPR measurements, the mAb supernatants were thawed and purified using a Cytiva ÄKTA pure 25 with Protein A HiTrap Fibro column (Cytiva 17549856). The eluates were controlled for quantity and integrity using SDS-Page gel electrophoresis under reducing and non-reducing conditions. The protein concentration was determined using the Qubit Protein Assay (Invitrogen Q33212) and 1 mg was further purified by size-exclusion chromatography with a Superdex 200 increase column. Aggregate-free fractions were pooled, concentrated and the concentration was determined by analysing the absorption at 280 nm and applying amino acid sequence-specific extinction coefficients. The samples were stored at 4 °C until the SPR measurements were performed.

For the *in vivo* protection analysis, supernatants were thawed and purified using 5 ml HiTrap Protein G HP affinity chromatography columns (Cytiva 17040503). To avoid endotoxin contamination, columns were used only once and all materials were applied to the columns by hand using single-use Luer Lock plastic syringes (Braun 461709F) and Luer Lock extension lines (Braun 4097173) under the laminar flow workbench at a flow rate of <5 ml/min. The mAbs were immediately dialysed twice against a 100-fold volume of PBS and sterile filtered. The protein concentration was determined via Qubit Protein Assay (Invitrogen Q33212) and the endotoxin content was verified to be <0.5 EU/mg the Chromogenic Endotoxin Quant Kit (Thermo Scientific A39552). Aliquots were stored at −80 °C until use.

### SARS-CoV-2 infection of hamsters

The animal experiment was conducted under veterinary observation and efforts were made to minimise distress. Antibody injection and virus challenge was performed under isoflurane anaesthesia, while euthanasia was conducted by administrating overdosed intraperitoneal injection of anaesthesia (for details see below). Approval to conduct the experiment was given by the German Niedersächsisches Landesamt für Verbraucherschutz und Lebensmittelsicherheit (LAVES file number 21/3755). Twenty-eight Syrian hamsters (*Mesocricetus auratus*, Janvier Labs), 6–10 weeks old, were housed under BSL-3 conditions, starting 10 days prior to the experiment, and randomised into 4 groups of 7 animals, each. The sample size was calculated using the Power analysis package ‘pwr’ in R using the ANOVA option and a preliminary effect size of 0.63, a significance level of 0.05 and a power of 0.8. Hamsters were injected intraperitoneally with 10 mg/kg of MB025_A07K, MB025_A09L, MB027_E04L, or a SARS-CoV-2 non-specific human IgG control mAb (Palivizumab) in a volume of 500 μl. Animal care takers as well as the pathologists who analysed the samples were blinded for the treatment groups. The hamsters were challenged intranasally with 10^4^ TCID_50_ of a SARS-CoV-2 614G isolate, 24 h after receiving the respective antibody.^11^ To verify the identity of the used virus strain, the whole viral genome was sequenced prior to the experiment and the corresponding spike nucleotide and deduced amino acid sequences are shown in Table S3. The animals were monitored daily for body weight loss and clinical symptoms until they were euthanised four days after infection. Hamsters were euthanised by applying an overdosed intraperitoneal injection of Ketamine-Medetomidine solution in sterile sodium chloride (100 mg/ml Ketamidor, WDT, Garbsen, Germany; 1 mg/ml Domitor, Orion-Pharma, Ismaning, Germany) with a dose adjusted for each hamster's weight (1000 mg/kg Ketamine, 1.25 mg/kg Medetomidine).

Infectious SARS-CoV-2 virus particles were quantified as previously described.^12^ In brief, lung and nasal turbinate tissues were collected after necropsy and homogenised using a TissueLyser II (Qiagen). Calu-3 cells (ATCC HTB-55) were infected with 10-fold serial dilutions of the homogenised tissue prepared in MEM + 2% FBS. Plates were further incubated in a humidified atmosphere, at 37 °C, 5% CO_2_. Five days after infection cells were fixed with 4% PFA and labelled using an anti-SARS-CoV-2 nucleocapsid antibody (SinoBiological). Virus titres (TCID50/ml) were calculated using the Spearman–Karber method.

### Histopathology and SARS-CoV-2 immunohistochemistry

The left lung lobe, trachea, and nose were collected in 10% neutral-buffered formalin (NBF; VWR International GmbH, Hannover, Germany). Prior to fixation the left lung lobe was injected with NBF according to described protocols.^13^ Noses were decalcified with ethylene-diamine-tetra-acetic acid for three weeks (Decalcifier soft, Carl Roth GmbH & Co. KG, Karlsruhe, Germany). All tissue sections were dehydrated, embedded in paraffin and trimmed (2–3 μm sections), mounted on glass slides and stained with haematoxylin and eosin according to routine protocols.

Histopathological assessment of the respiratory tract was performed by two veterinary pathologists (WR, WB) using a semiquantitative scoring system according to routine protocols with minor modifications.^11^ Briefly, alveolar, vascular, and conductive airway lesions were evaluated in the lungs. In addition, changes in the trachea as well as in the nasal respiratory and olfactory epithelium were evaluated as previously described.^11^ Total scores were generated by multiplying extent and severity of nasal, tracheal, and pulmonary lesions before adding up all scores.

SARS-CoV-2 nucleoprotein immunohistochemistry was performed according to established protocols.^11^ Sections were deparaffinized, rehydrated and incubated with a mouse monoclonal antibody against SARS-CoV-2 nucleoprotein (Sino Biological, Peking, China-40143-MM05). For negative controls, the antibody was replaced by respective protein concentration of ascitic fluid from non-immunised BALB/cJ mice. The immunoreaction was visualised using the EnVision+ polymer system (Dako Agilent Pathology Solutions) and 3,3′-diaminobenzidine tetrahydrochloride as chromogen (Sigma Aldrich).

All slides were evaluated with a Zeiss Axioscope (Zeiss, Göttingen, Germany; field of view at 400× magnification: 0.16 mm^2^). Whole slides were scanned with the Olympus VS200 slide scanner (Olympus Deutschland GmbH, Hamburg, Germany) and images of animals representing group median score were exported with the respective OlyVIA software.

### Virus antibody escape assay

The protocol for generation of virus escape mutants was adapted from Baum *et al*., 2020.^14^ Briefly, supernatant containing the monoclonal antibodies of choice were quantified using an IgG1 ELISA (Invitrogen BMS2092) and six decreasing concentrations of the antibodies were incubated with 0.5 × 10^6^ pfu of recombinant, replication competent, GFP^+^ VSV∗ΔG-SARS-CoV2-SΔ21^15^ (hereafter referred to as VSV∗ΔG-S) in 24-well plates for 1 h using a total culture volume of 1 ml per well (“passage 1”). The mixture was transferred to Vero-B4 cells (DSMZ ACC 033) and incubated for 4 days. From the well with visible fluorescence under the highest mAb concentration, the supernatant containing the virus was distributed to six new wells in a 1:10 dilution with fresh antibody dilutions (“passage 2”). In case fluorescence was observed in the wells with the highest antibody concentration in passage 2, we presumed that the virus had escaped from neutralisation.

### Sequencing of VSV∗ΔG-S escape mutants

Viral RNA was isolated from VSV∗ΔG-S virus mutant strains using a MiniPrep Plus Kit (Zymo R2070) and cDNA was generated using the PrimeScript RT Master Mix (Takara RR0364). Since all tested neutralising mAbs were verified to bind to the SARS-CoV-2 spike RBD only, we amplified this region via polymerase chain reaction (PCR) using the primers VSV_SARS2_RBD_fw GCTCGTGCTGCAGCTTATTA and VSV_SARS2_RBD_rev AAACACGCCAAGTAGGAGTAAG. The derived amplicons were purified using PCR cleanup (Qiagen 28506) and sequenced via Sanger sequencing in 2 separate reactions in the forward and reverse directions (Eurofins Genomics). The Sanger sequencing data were processed to detect SNP using the tool Indigo^16^ (www.gear-genomics.com). SnapGene 7.1 was used to visualise the sequence traces and identify the consequential amino acid substitutions.

In addition, the amplicons were sequenced via Oxford Nanopore sequencing (Plasmidsaurus). The quality of the fastq files was assessed using FastQC (version 0.11.9). Low-quality reads and bases were removed using NanoFilt (version 2.6) by applying an average quality score threshold of 10, a read length of 650 bases, and by trimming the first 10 bases of each read. For additional correction of the reads, the raw reads were hierarchically assembled using Canu (version 2.0). Subsequently, minimap2 (version 2.24) was employed to align the filtered reads against the assembled reads. Minimap2 was also used for the realignment of the corrected reads against a customised RBD reference from R319-F541 of the SARS-CoV-2 spike protein, including an A372T substitution, which emerged during cell culture adaptation.

To obtain statistics for the nucleotides in aligned sequences, the basecounts tool (version 0.1) was used. Additionally, during the analysis, Samtools (version 1.11) was employed for sam-to-bam conversion, sorting, and indexing. Picard (version 3.1) was used for converting sam to fastq format and Racon (version 1.5) for creating a consensus fasta file from the mapped reads. An automated workflow of the analysis was generated using Snakemake (version 7.32.3) and has been provided on github.com/memumab/escapster.

For further statistical analysis and visualisation, the sequences were imported into R (version 4.3.1). The variable nucleotides were classified into synonymous or non-synonymous mutations. A heatmap highlighting the detection frequency of nucleotides was plotted using ComplexHeatmap package (version 2.16).

For the sequencing of SARS-CoV-2 genomes from the *in vivo* challenge experiment, RNA was extracted as described above and cDNA was generated using ProtoscriptII reverse transcriptase enzyme (New England, BiotechnologyBioLabs) according to the manufacturer's protocol. For nanopore sequencing, primers were designed as described previously, with amplicon length set to 500 base pairs and a 75 base pair overlap between amplicons.^17^ Libraries were generated using native barcode kits from nanopore (EXP-NBD104, EXP-NBD114 and SQK-LSK109). Sequencing was performed on a R9.4 flow cell. The resulting data was processed as described above, but with a modified reference sequence that did not contain the A372T substitution.

### Surface plasmon resonance measurements

For SPR measurements, a Biacore 8K+ instrument was used with a C1 sensor chip. Four antigens were immobilised simultaneously, SARS-CoV-2 RBD of the Wuhan strain (Acro Biosystems SPD-C82E9-25), the VOC Alpha (SPD-C82E6-25), Delta (SPD-C82Ed-25), and Omicron (SPD-C82Eq-25). Diluted mAbs served as the analytes in 10 mM HEPES pH 7.4, 150 mM NaCl, 3 mM EDTA, and 0.05% Tween 20 analysis buffer. First, a qualitative analysis was performed at 25 °C as pilot experiment to determine the optimal concentration range. Afterwards, quantitative measurements were performed at 37 °C with a flow-rate of 50 μl/min over a range of at least 8 concentrations. Subsequently, k_a_ [M^−1^s^−1^], k_d_ [s^−1^], K_D_ [M], & R_max_ [RU] values were calculated using the Biacore Insight software. The SPR traces were plotted using GraphPad Prism 10.

Epitope binning was performed on the Wuhan RBD using a flow rate of 10 μl/min by first saturating the antigen-coated chip with one mAb and sequentially testing all other mAbs for their ability to bind the same antigen in addition to the first mAb. Ultimately, 9 antibodies were tested against each other and non-overlapping epitopes were defined based on the competition data.

### Determination of Fc-mediated effector functions

Monoclonal antibodies (mAbs) were used in a 4-fold serial dilution from 0.024 μg/ml to 100 μg/ml. Cocktails of antibodies were prepared at a total concentration of 100 μg/ml. ADCD and ADCP were carried out in technical replicates. Neutrophils and NK cells from two healthy donors were separately procured to acquire biological replicates for ADNP and ADNKA, respectively. The respective assay buffers served as negative controls while human serum from a recent symptomatic COVID-19 patient was used as positive control.

Recombinant trimeric spike of wild-type SARS-CoV2 (D614G) (Sino Biological Inc., China) was biotinylated using the NHS-Sulfo-LC-LC kit (Thermo Fisher, MA, USA) according to the manufacturer's instructions. Excessive biotin was removed using Zeba spin desalting columns with a 7 kDa cut-off (Thermo Fisher). NeutrAvidin-labelled 1.0 μm microspheres (Invitrogen, MA, USA) were incubated with 10 μg of biotinylated antigen for 2 h at 37 °C.

### Antibody-dependent complement deposition (ADCD)

Antigen-specific mAb-induced deposition of complement was assayed as detailed previously.^18^ Antigen-coated non-fluorescent beads were combined with mAbs or cocktails for 2 h at 37 °C. Guinea-pig complement (Cedarlane, ON, Canada) was diluted in GVB++ buffer and transferred to the pre-formed immune complexes. After 20 min incubation at 37 °C and 5% CO_2_, the reaction was stopped with PBS containing 15 mM EDTA. Surface-deposited C3 was labelled with 1:100 diluted anti-guinea pig C3a-FITC antibody (MP Biomedicals, CA, USA) and samples were fixed with 4% paraformaldehyde solution. The mean-fluorescent intensity (MFI) of C3 was measured on an ID7000 Spectral Cell Analyser (Sony Biotechnology).

### Antibody-dependent neutrophil-phagocytosis (ADNP)

Level of mAb-stimulated phagocytosis by neutrophils was investigated as described previously.^19^ First, mAb cocktails or single mAbs were incubated with antigen-coated yellow-green fluorescent beads for 2 h at 37 °C. Neutrophils were isolated from whole blood by erythrocyte lysis with Ammonium–Chloride–Potassium (ACK) buffer (Biomol, Germany). Immune complexes were then incubated with 50,000 neutrophils in R10 media (RPMI 1640, 10% FCS, 1 × GlutaMax, 1 × Penicillin-Streptomycin, 10 mM HEPES) for 1 h at 37 °C and 5% CO_2_. To define neutrophils, the cells were labelled with 1:100 diluted anti-human CD66b-Pacific Blue antibody (BioLegend, CA, USA, RRID: AB_2563294). Once fixed with 4% PFA for 20 min, the phagocytic activity was determined on an ID7000 Spectral Cell Analyser (Sony Biotechnology) and described as the product of frequency and MFI of bead positive neutrophils (phagocytosis score).

### Antibody-dependent THP-1 cell-phagocytosis (ADCP)

The capacity of mAbs to induce phagocytic activity in THP-1 monocytes was assessed as reported before.^20^ Briefly, immune complexes were formed by incubation of mAbs or cocktails with antigen-coated yellow-green fluorescent beads for 2 h at 37 °C and 5% CO_2_. Once washed, complexes were combined with 25,000 cultured THP-1 monocytes (#ACC 16, DSMZ, Germany) and placed overnight at 37 °C and 5% CO_2_. Before measurement on an ID7000 Spectral Cell Analyser (Sony Biotechnology), cells were fixed for 20 min with 4% PFA. Similar as for the ADNP, phagocytic activity was captured as phagocytosis score.

### Antibody-dependent NK cell-activation (ADNKA)

Activation of NK cells by mAbs was examined. A 96-well flat bottom high-binding plate was coated with 50 μl (3 μg/ml) spike trimer of wild-type SARS-CoV2 (D614G) overnight at 4 °C. Following blocking with 5% BSA-PBS for 2 h, plates were incubated with mAbs or cocktails overnight at 4 °C. NK cells were isolated from leucocyte reduction cones of aphaeresis donations from healthy donors using the RosetteSep NK cell enrichment Cocktail (STEMCELL Technologies, MA, USA) according to the manufacturer's instruction. Purified NK cells were incubated in R10 media with 1 ng/ml of recombinant human IL-15 (BioLegend) overnight at 37 °C and 5% CO_2_. Plates were washed and incubated with 50,000 NK cells along with 2 μg Brefeldin A (Sigma–Aldrich), 1:80 GolgiStop (BD Biosciences, NJ, USA) and 1:11 diluted anti-human CD107a-BV605 (BioLegend, RRID: AB_2563851) for 5 h at 37 °C and 5% CO_2_. Next, 1:40 diluted anti-human CD3-APC-Cy7 (BioLegend, RRID: AB_830755) and 1:20 diluted anti-human CD56-PE-Cy7 (BioLegend, RRID: AB_2857328) were added for 15 min. Cells were fixed and permeabilized with FIX & PERM (Invitrogen) according the manufacturer's protocol and labelled intracellularly with 1:200 anti-human MIP-1β-BV421 (BD Biosciences, RRID: AB_2737877) and 1:50 anti-human IFN-γ-PE (BioLegend, RRID: AB_315440) for 15 min. After a final wash, the MFI of extracellular and intracellular activation and degranulation markers was acquired on an ID7000 Spectral Cell Analyser (Sony Biotechnology).

### Statistical methods

For comparison of virus titres and pathology scores between the experimental groups in the animal experiment, groups were analysed by Kruskal–Wallis multi-comparison test with Dunn's correction for multiple comparisons in GraphPad Prism 10. Exact p values ≤ 0.05 were assumed statistically significant. Before non-parametric testing, non-normality was ascertained using the Kolmogorov–Smirnov test. Correlation analysis of biological replicates in the *in vitro* viral escape assay was performed using linear regression analysis in GraphPad Prism 10.

### Role of funders

The Funders of this study had no role in the study design, data collection, data analyses, interpretation or writing of the report.

## Results

### Hamsters adoptively transferred with SARS-CoV-2 neutralising mAbs sporadically show breakthrough-infections

**Fig. 1:**
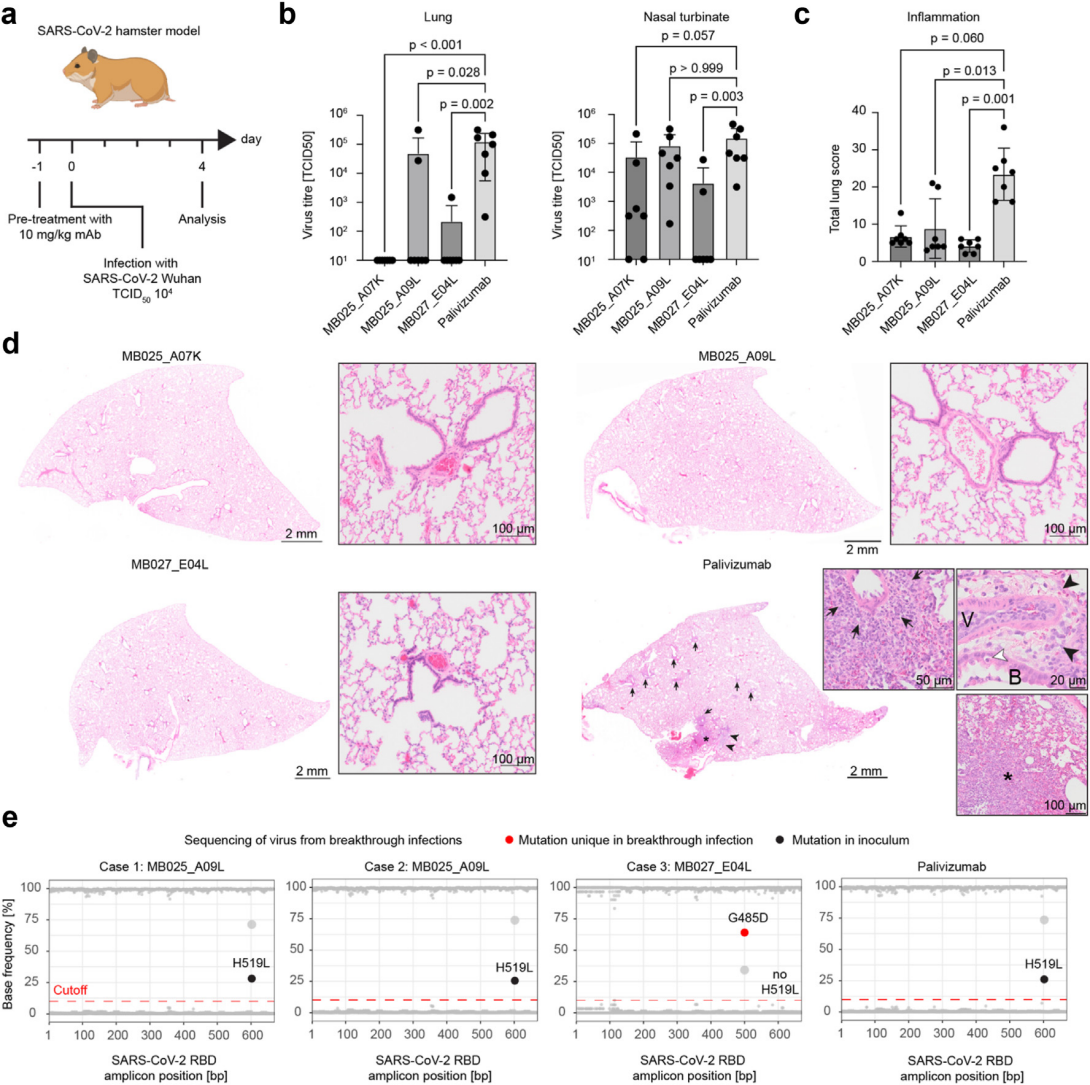
**Hamsters treated with SARS-CoV-2 neutralising mAbs and subsequently infected with SARS-CoV-2 occasionally show breakthrough infections. a)** Schematic depiction of the experimental outline. **b)** Virus titres in lungs and nasal turbinates of SARS-CoV-2 infected animals at 4 dpi. **c)** Total lung scores for pathomorphological changes in hamsters at 4 dpi. **d)** Pathomorphological changes of hamster lungs. Representative images of one animal per treatment group (median group score) are shown at 4 dpi. The treatment group is indicated above. Inserts represent higher magnifications of the respective lungs. While no or minimal lesions were identified in all three mAbs treatment groups (MB025_A07K, MB025_A09L, MB027_E04L), multifocal atelectasis (asterisk) with prominent perivascular and peribronchial inflammation (arrows) as well as perivascular oedema (arrow heads) was present in the Palivizumab treated hamsters (V: vessel, B: bronchiole). Haematoxylin and eosin staining. **e)** SARS-CoV-2 genome sequencing data for the three breakthrough infections and an infected animal from the control group (Palivizumab). At each nucleotide position (X-axis), the plot shows the frequency of A, T, G, and C as detected by Oxford Nanopore sequencing. The cutoff of 10% was chosen to discriminate sequencing noise from specific signal. For non-synonymous mutations, the consequential amino acid exchange is indicated as dot label. The mutation G485D is highlighted in red.

We previously described a set of SARS-CoV-2 spike-specific mAbs that were isolated from COVID-19 convalescent individuals early during the pandemic.^7^ Based on criteria that are critical for the clinical development of mAbs, including specific antiviral activity, neutralisation potency, maximal *in vitro* expression levels, and the tendency to form mAb aggregates, we selected the three most promising mAb candidates, MB025_A07K, MB025_A09L, and MB027_E04L (the latter being a broadly neutralising mAb against several SARS-CoV-2 VOC including Omicron BA.1, BA.2, and BA.3, but not BA.4/5). We further tested the protective capacity of these three mAbs *in vivo* in the hamster model.^11^ Injection-grade material was produced in serum-free ExpiCHO cells and mAbs were purified by affinity chromatography. Each mAb, including the SARS-CoV-2 non-binding and RSV-neutralising mAb Palivizumab that was used as a negative control, was applied intraperitoneally at a dose of 10 mg/kg to seven animals. 24 h post treatment, the animals were infected intranasally with the Wuhan strain of SARS-CoV-2 (614G)^11^ and sacrificed 4 days later for analysis of viral titres and airway pathology (Fig. 1a). All three SARS-CoV-2 mAbs were able to significantly reduce the virus titres in the lung of the animals (p < 0.001 for MB025_A07K, p = 0.028 for MB025_A09L, and p = 0.002 for MB027_E04L), while the extent of protection in the nasal turbinate was variable (Fig. 1b). The scores of lung pathology reflected the overall picture with reduced scores (p = 0.060 for MB025_A07K, p = 0.013 for MB025_A09L, and p = 0.001 for MB027_E04L) in all three test groups (Fig. 1c). Pre-treatment with all three mAbs was able to suppress the formation of lung atelectasis, inflammation, and oedema when compared with Palivizumab treated animals (Fig. 1d). Although the mAbs were highly efficacious, we observed that three out of 21 animals (14.3%) developed breakthrough infections, which presented with virus titres in the lung that reached similar levels as detected in the control group. We hypothesised that the virus stochastically mutated and that viral immune escape variants replicated in the presence of the administered mAbs. Sequence analysis of the lung homogenates revealed the presence of the non-synonymous H519L spike mutation that was identified in multiple mAb-treated animals as well as in the placebo control group. Therefore, this H519L substitution presumably was present already in the original virus preparation at low frequency. In contrast, sequence analysis of lung homogenates from the MB027_E04L treated animal that showed a breakthrough infection revealed a G485D mutation in the majority of the sequencing reads that was not present in the control group and therefore presumably was a treatment-induced antibody escape mutant (Fig. 1e). This observation supports the conclusion that viral escape mutations are selected in mAb treated hamsters and that the propensity of SARS-CoV-2 to escape from neutralisation by mAbs can be addressed in this *in vivo* model.

### Somatic hypermutations of mAbs affect the spike positions that are preferentially mutated during viral escape

**Fig. 2:**
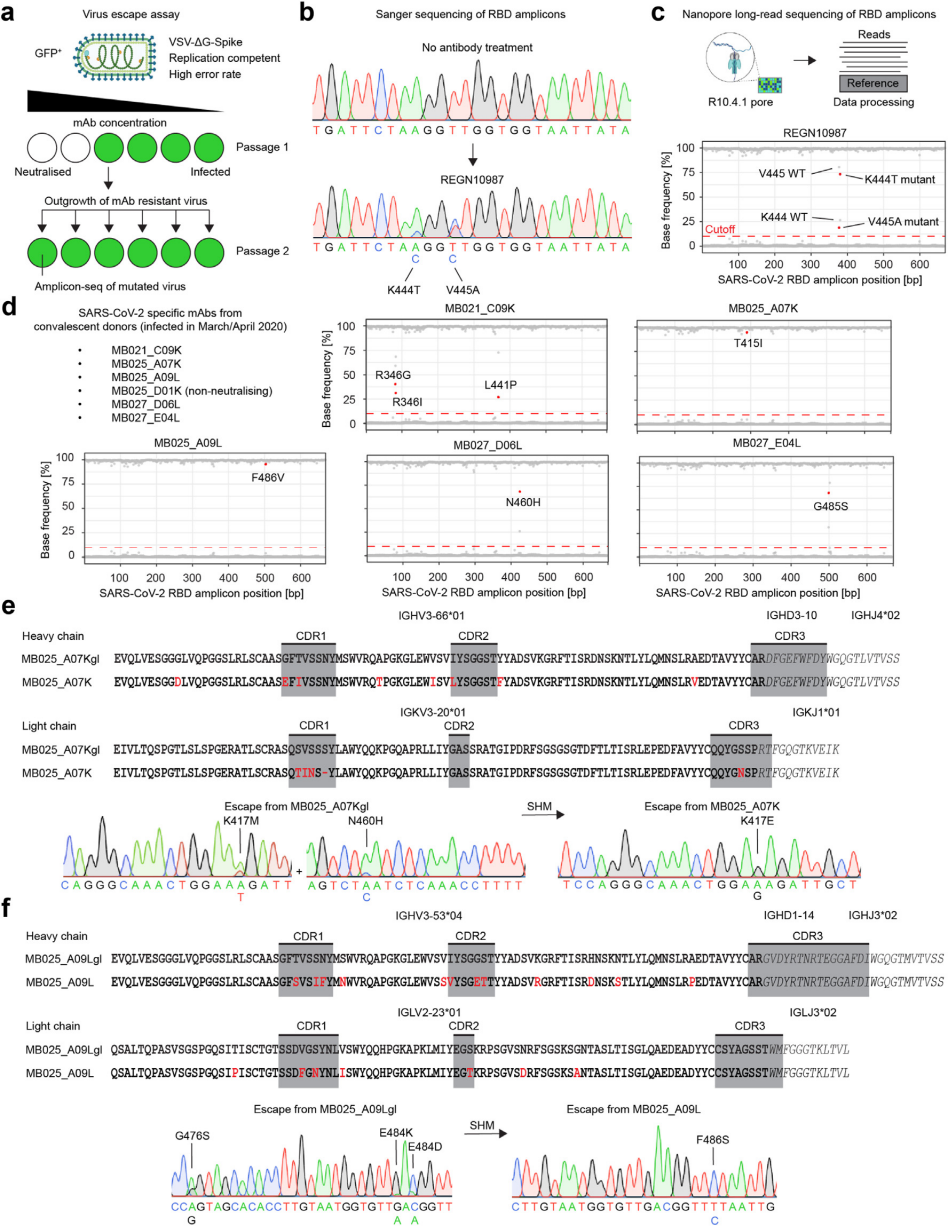
**Fast *in vitro* viral antibody escape assay reveals spike mutations that prevent mAb binding. a)** Experimental outline: Recombinant, replication-competent VSV∗ΔG-S was used to select escape mutants in the presence of mAbs. **b)** Exemplary result for the Sanger sequencing of untreated virus (upper panel) and virus treated with a control mAb REGN10987 (lower panel). **c)** Mutation frequency determination of control mAb REGN10987 using a customised Oxford Nanopore sequencing pipeline. At each nucleotide position (X-axis), the plot shows frequencies of A, T, G, and C as detected by Oxford Nanopore sequencing. The cutoff of 10% was chosen to discriminate sequencing noise from specific signal. For non-synonymous mutations, the consequential amino acid exchange is indicated as dot label. **d)** Sequencing results of escape mutants that were generated against a panel of SARS-CoV-2 neutralising mAbs which were described previously. **e)** Somatic hypermutation analysis of mAb MB025_A07K and determination of viral escape mutants selected against the germline and somatically hypermutated mAb versions. V gene segments are indicated in regular fonts with SHM highlighted in red. D and J gene segments are indicated by italic fonts, no SHM were altered in these regions. Complementarity-determining regions (CDR) as determined by IgBlast are highlighted in grey boxes. **f)** Similar analysis as in e) for the mAb MB025_A09L.

To test viral escape from mAbs *in vitro*, we utilised replication-competent recombinant vesicular stomatitis virus (VSV), in which the endogenous glycoprotein was genetically replaced by the SARS-CoV-2 spike protein (VSV∗ΔG-S).^15^ Because VSV mutates more rapidly than SARS-CoV-2 and does not require as stringent safety precautions as clinical SARS-CoV-2 isolates, this model allows fast generation of viral escape mutants. We established an assay in which escape mutants were selected in the presence of graded concentrations of neutralising mAbs. Following RNA isolation and cDNA synthesis, the RBD region of the spike was amplified by PCR and sequenced (Fig. 2a). In order to identify spike mutations that caused resistance to neutralisation by the respective mAbs, we sequenced the amplicons by using Sanger sequencing and Oxford Nanopore long-read sequencing. We validated the system using REGN10987, which was previously shown to select for mutations at spike positions K444 and V445.^14^ Indeed, in our assay spike escape mutations appeared at the same positions in the Sanger chromatograms (Fig. 2b) as well as in the base-calling graphs after mapping the Nanopore long-reads to the spike reference sequence (Fig. 2c).

In this assay, we analysed a set of five recombinant virus neutralising mAbs that were derived from V(D)J sequencing of memory B cells of convalescent individuals, three of which were tested in the hamster model above. Distinct viral escape mutations were sequenced for each of the five mAbs. Importantly, the *in vitro* escape study revealed a spike escape mutation against MB027_E04L at the same G485 position as detected in the hamster model (Fig. 2d).

To further study viral escape by germline vs. hypermutated mAbs, we synthetically generated close-to-germline versions of the mAbs MB025_A07K and MB025_A09L^7^ and analysed these mAbs and their original counterparts in our *in vitro* viral escape assay. The retrieved data indicated that the germline mAb MB025_A07Kgl triggered viral escape at the spike positions K417M and N460H, while a K417E mutation was detected in presence of the matured mAb (Fig. 2e and Table S2) and for MB025_A09L, SHM shifted viral escape from G476S, E484K, and E484D in the presence of the germline mAb towards F486S in the presence of the matured mAb (Fig. 2f).

### Germline antibodies bind the spike protein with up to nanomolar affinity

**Fig. 3:**
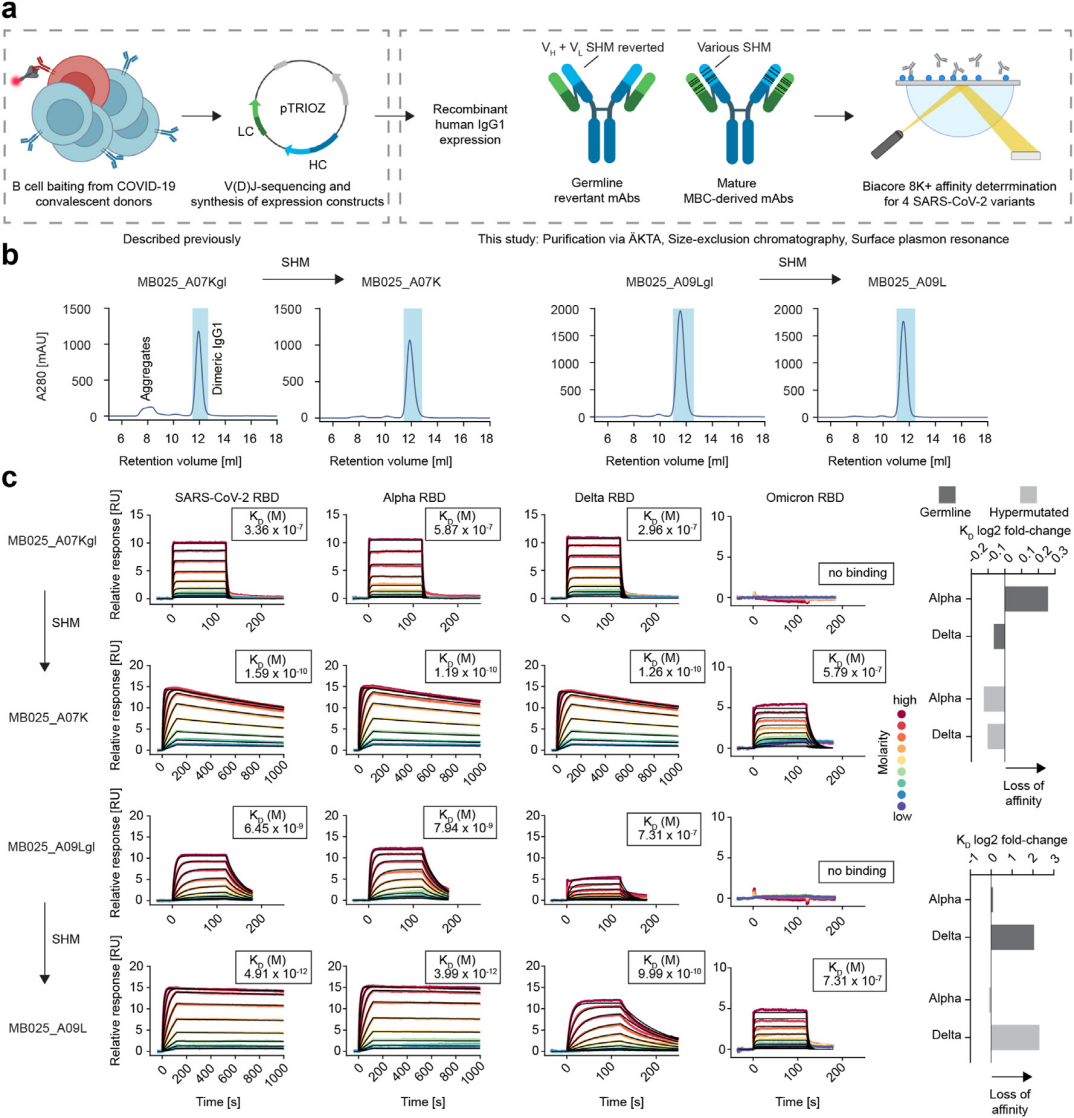
**The germline versions of the analysed mAbs retain their affinity to VOC to different degrees. a)** Schematic depiction of the experimental outline. **b)** Isolation of aggregate-free recombinant IgG1 via SEC. **c)** Quantitative SPR measurements of MB025_A07K and MB025_A09L in comparison with their germline versions. The four columns represent four antigen variants to be immobilised on the chip. Log2 fold-changes in affinity normalised to the wild-type SARS-CoV-2 RBD are plotted on the right side.

In addition to modulating the positions in the SARS-CoV-2 spike protein that conferred escape from neutralisation *in vitro*, the reversion of the SHM in mAbs to germline furthermore revealed that some of the analysed mAbs exerted potent neutralising activity already in their germline configuration, but SHM was required for the cross-neutralisation of VOC such as Alpha and Delta.^7^ In order to achieve a more precise understanding of the effect of SHM on the binding affinity of these mAbs to the RBD of different SARS-CoV-2 VOC, we produced larger quantities of six mAbs that we published earlier^7^ and of the germline versions of MB025_A07K and MB025_A09L. These mAbs were purified by affinity chromatography (Fig. S1), followed by size-exclusion chromatography (SEC), and the affinity of the mAbs was determined for four SARS-CoV-2 VOC by surface plasmon resonance (SPR) measurements (Fig. 3a).

SEC allowed the removal of mAb aggregates, which were observed for some mAbs, such as MB025_A07Kgl (Fig. 3b). Certain mAbs showed strong binding affinity towards all VOC, while others failed to bind certain VOC, e.g., MB027_D06L did not bind the Omicron variant, or the mAbs showed reduced affinity towards Alpha and Delta (full dataset in Fig. S2). As expected, the comparison of binding affinity of germline vs. hypermutated mAb versions revealed a strong affinity increase upon introduction of SHM, although the germline version of MB025_A09L bound the antigen already with single-digit nanomolar affinity and the binding further improved by more than 1000-fold, resulting in a low picomolar range of affinity upon inclusion of SHM (Fig. 3c). In addition, we observed a moderately reduced binding affinity of the germline mAb MB025_A07Kgl towards Alpha (K_D_ = 3.36 × 10^−7^ M for WT vs. 5.87 × 10^−7^ M for Alpha), which was not observed for the mature mAb and may explain the reduced ability of MB025_A07Kgl to neutralise the Alpha variant. A strongly reduced affinity of MB025_A09Lgl towards Delta (K_D_ = 6.45 × 10^−9^ M for WT vs. 7.31 × 10^−7^ M for Delta) was observed, confirming the inability of MB025_A09Lgl to neutralise the Delta variant.^7^ Notably, the hypermutated mAb MB025_A09L also bound the Delta RBD with massively reduced affinity when compared with the wild-type RBD, but the reduced affinity was still in the nanomolar range and thus sufficed to efficiently neutralise the virus. The Omicron variant was only bound by the hypermutated, but not by the germline mAbs (Fig. 3c). Overall, these newly generated SPR data confirmed our previous results that we retrieved in virus neutralisation assays with the same mAbs.^7^ Furthermore, we conclude that the previously observed increase in neutralisation breadth by SHM could be due to direct enhancement of the binding affinity specifically against a certain variant, or an indirect enhancement against all variants, both leading to similar outcomes.

### B-cell clonotype diversification counteracts viral escape

**Fig. 4:**
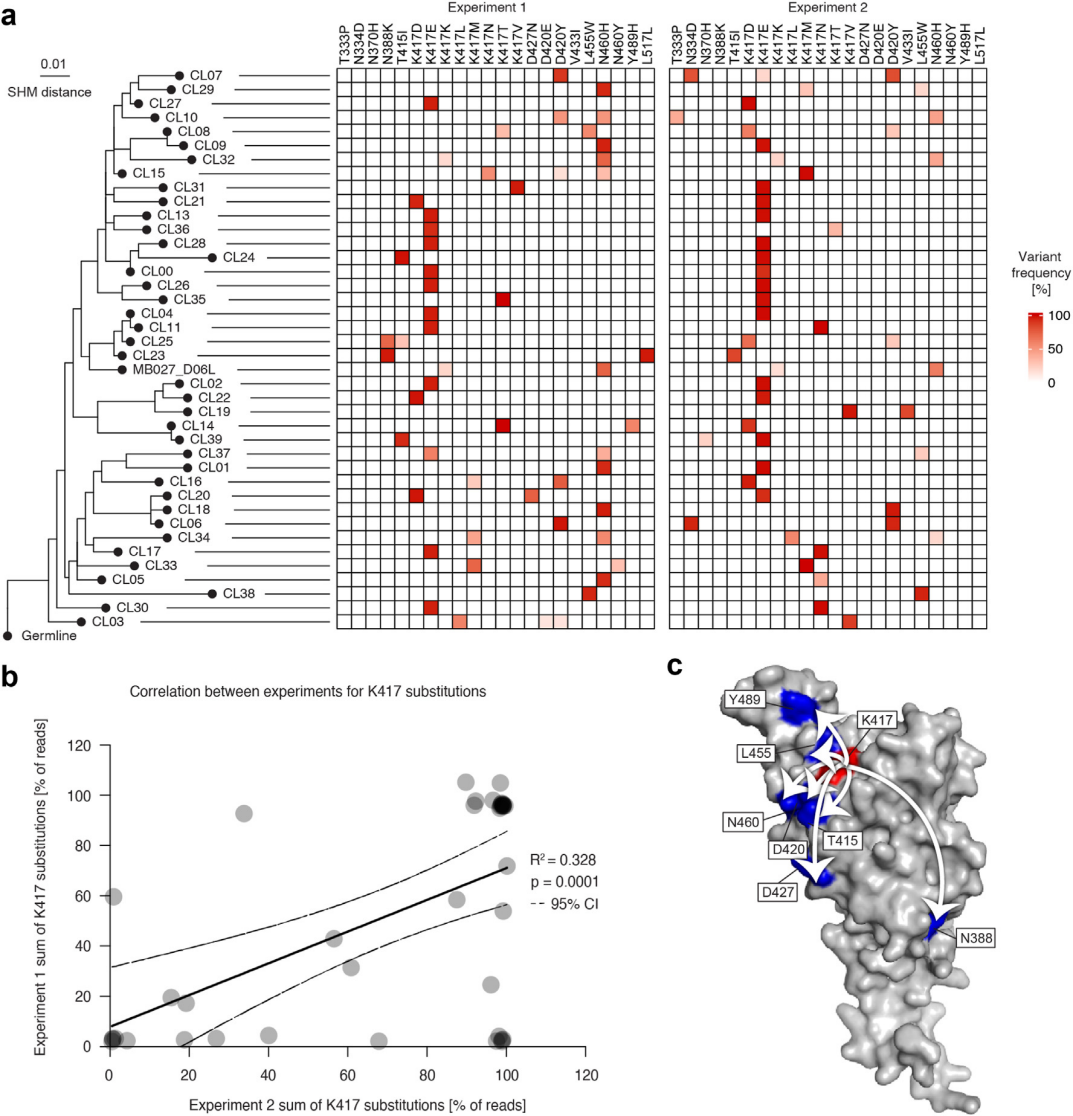
**Stochastic clonotype diversification of B cells by somatic hypermutation shifts the viral escape profile against single mAbs. a)** Phylogenetic tree of an immunodominant clonotype (as determined previously by scRNA-seq^10^). The heatmaps indicate the presence and frequency of viral escape mutants in the spike protein on the amino acid level after mAb treatment. Two independent experiments were performed. **b)** Correlation analysis between the two experiments for the presence of mutations in the spike K417 amino acid locus. The line represents a linear regression analysis with 95% confidence intervals plotted as dashed lines. **c)** Spatial arrangements of the identified escape hotspots on the SARS-CoV-2 RBD. Arrows symbolise alternative escape mutations (blue) for K417 (red).

As SHM of antibodies affects the selection of viral escape mutations, we next aimed to substantiate our findings using a set of 40 clonally related (CL) mAbs, which all differed in the number, position, and combination of SHM. We showed earlier that some members of this clonotype neutralised the VOC Beta and Gamma. MB027_D06L, which was identified in the same blood donor after recovery from COVID-19 and before vaccination, was unable to neutralise the VOC Beta and Gamma.^10^ The analysis of all the antibodies in the clonotype revealed that the preferred viral escape mutations followed a pattern, in which the less mutated mAbs induced escape in the spike protein at position N460, the moderately mutated ones at position K417, while the clones with enhanced numbers of SHM preferentially induced spike escape again at amino acid position N460. Some clones differed from this pattern and typically induced viral escape at positions N388, T415, D420, D427, L455 or Y489, but not at positions N460 or K417 (Fig. 4a). Since viral escape is a stochastic process with viral mutants occurring in a random manner, whereas subsequently neutralisation resistant virus variants are selected by the mAbs, we investigated to what extent the observed viral escape patterns are detected in independent experiments (Fig. 4a). The appearance of the position K417 in the spike protein of escape variants was selected by several mAbs and was detected in two independent experiments with significant (p = 0.0001) correlation (Fig. 4b). Spatially, all amino acids (except for N388) are part of the presumed epitope of the clonotype. Since we previously observed that some members of the clonotype gain the ability to neutralise the Beta and Gamma variants, which harbour a K417N or K417T mutation, respectively, we conclude that in these cases SHM can lead to a shift in the preferred viral escape mutations away from K417 towards N388, T415, D420, D427, L455 or Y489 (Fig. 4c). This would ultimately lead to a stochastic enhancement of the neutralisation breadth against VOC such as Beta and Gamma.

### Vaccination of a convalescent individual enhances the antibody affinity

**Fig. 5:**
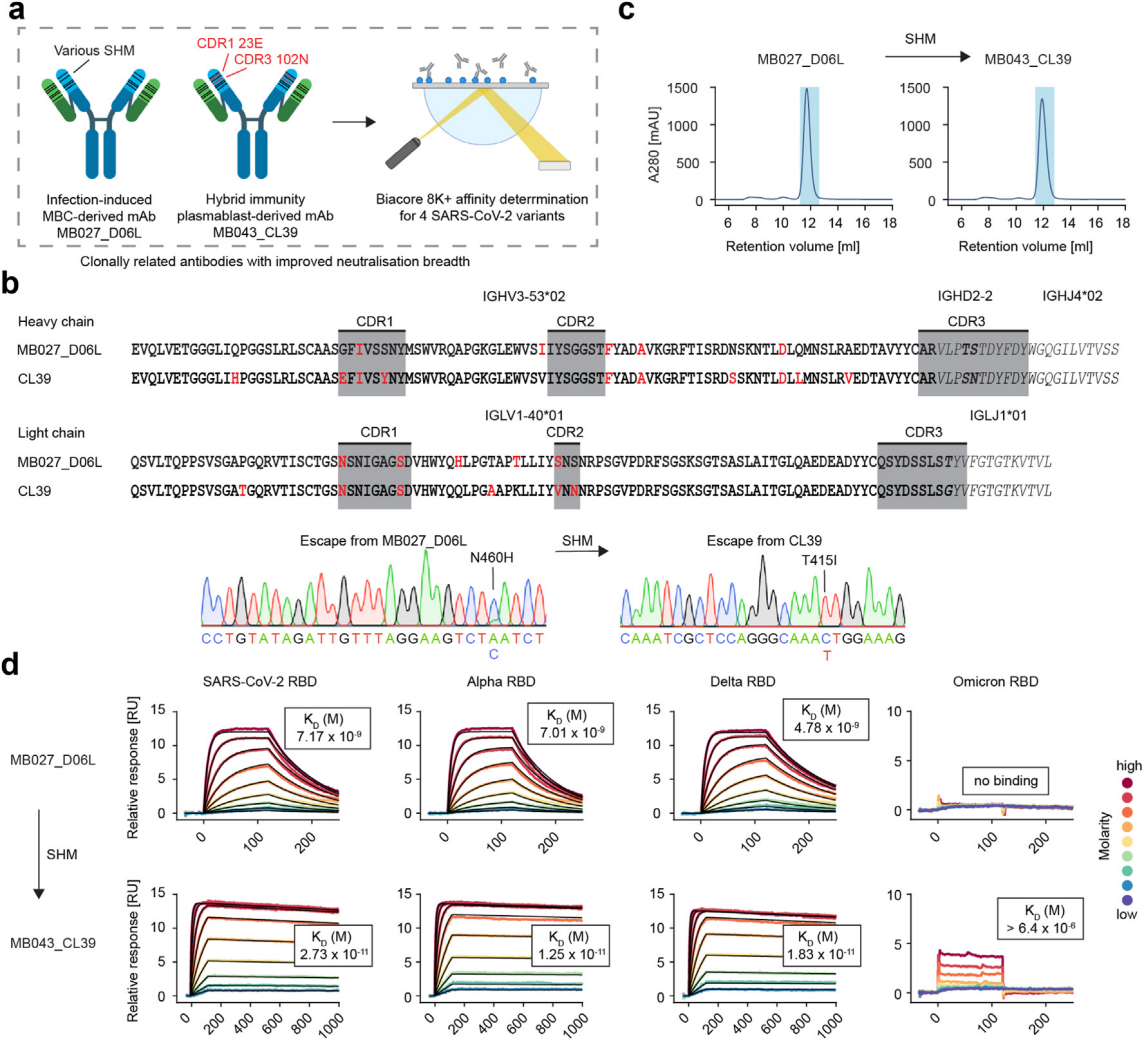
**Hybrid immunity enhances the affinity of SARS-CoV-2 neutralizing mAbs. a)** Schematic depiction of the experimental outline. **b)** Somatic hypermutation analysis of MB027_D06L and CL39 and the effect of differential hypermutation on viral escape. **c)** SEC for isolation of aggregate-free mAb peaks. **d)** Quantitative SPR measurements of MB027_D06L (infection-induced) and MB043_CL39 (same clonotype but sequenced from vaccine-induced plasmablast). The four columns represent four antigen variants immobilised on the chip.

The mAb MB027_D06L is derived from a memory B cell that was isolated from a donor after recovery from SARS-CoV-2,^7^ while many clones from the clonotype described above came from vaccine-induced plasmablasts from a blood donor who was vaccinated with a vaccine that encoded the spike of the same ancestral SARS-CoV-2 strain that also caused the infection.^10^ Notably, CL39 cross-neutralised the VOC Beta and Gamma as well as the ancestral SARS-CoV-2 strain, while MB027_D06L failed to neutralise these virus variants under all tested concentrations. In contrast to the germline versions of MB025_A07K and MB025_A09L described above, this effect was not detected upon reversion of SHM to germline, but the enhancement was detected in the clinically relevant setting of a (booster) vaccination. Therefore, we tested the affinity of the authentic mAbs MB027_D06L and CL39 by using SPR (Fig. 5a). The sequence alignment indicated that CL39 mainly contained additional SHM when compared with MB027_D06L. These differential SHM resulted in a change of preferred escape mutations from N460H for MB027_D06L towards T415I for CL39 (Fig. 5b and Table S2). No tendency of mAb aggregation was observed for these two candidates (Fig. 5c). Examining the binding of two related mAbs by SPR revealed that the affinity of the vaccine-induced mAb CL39 was more than 200-fold higher than that of the infection-induced MB027_D06L, and a moderate reactivity towards the Omicron RBD was newly gained (Fig. 5d). In conclusion, the presence of SHM correlates with the affinity of antibodies not only when artificial germline versions of the mAbs are compared with authentic mAbs, but also in clonally related mAbs that contain different SHM. This indicates that affinity maturation during convalescence can result in antibodies with enhanced affinity, increased neutralisation breadth, or a mixture of both.

### Simultaneous binding of mAbs suppresses viral escape and potentiates effector functions

**Fig. 6:**
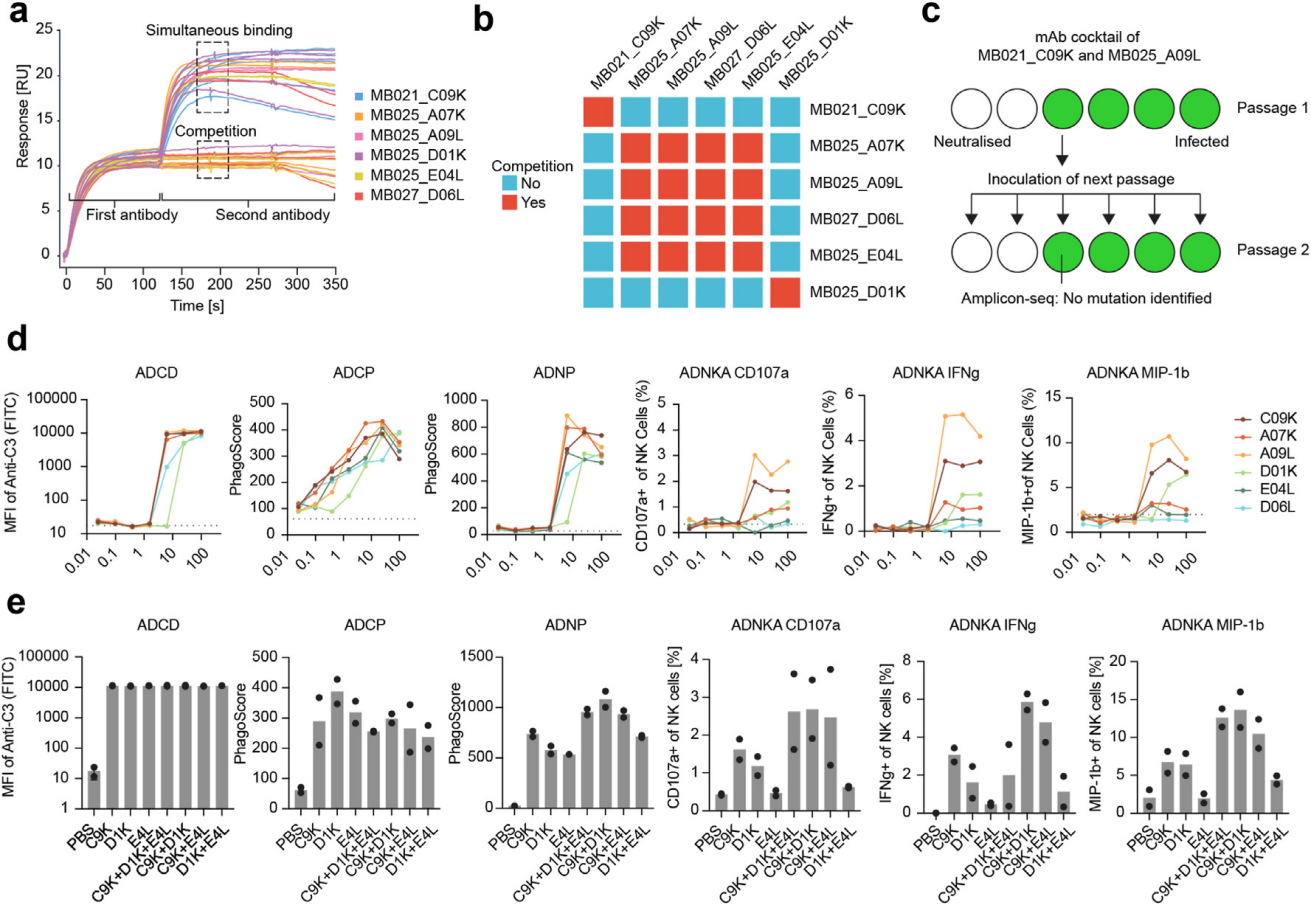
**Epitope binning analysis informs on mAb combinations that optimally avoid the induction of viral antibody escape variants. a)** Pairwise competition measurement using SPR. **b)** Epitope binning from pairwise competition measurement. Blue colour symbolises the ability of a mAb pair to bind the antigen simultaneously, red colour indicates competition of a mAb pair for their epitope on the antigen. **c)** Virus escape assay using VSV∗ΔG-S in presence of the mAb cocktail MB021_C09K and MB025_A09L. **d)** Dose-dependent measurement of Fc-mediated effector functions (Antibody-dependent complement deposition [ADCD], cellular phagocytosis [ADCP], neutrophil phagocytosis [ADNP], and NK cell activation [ADNKA]) for the six mAbs in the epitope binning analysis. **e)** Effect of cocktail formulation on the Fc-mediated effector functions.

It was previously shown that pairwise combination of mAbs with non-overlapping epitopes can effectively suppress the induction of viral escape.^14^ To define suitable candidates for a mAb cocktail that suppresses viral escape, we performed epitope binning using SPR, in which each antibody from our previously identified collection was tested for epitope competition against another antibody from the collection. By saturating the antigen-coated chip with the first mAb and subsequently loading the second mAb, we were able to distinguish mAbs that compete for the same epitope and mAbs that can simultaneously bind, i.e., that bind different epitopes of the SARS-CoV-2 RBD (Fig. 6a). The analysis indicated the presence of three non-overlapping epitopes, with MB021_C09K and MB025_D01K binding to different epitopes, and the four other mAbs, including the three mAbs tested *in vivo* (see above), binding to the same epitope (Fig. 6b). We hypothesised that the neutralising mAb MB021_C09K could be combined pairwise with a neutralising mAb from the alternative epitope in an escape-suppressing cocktail. Indeed, when we formulated a 1:1 mixture of MB021_C09K and MB025_A09L, the formation of viral escape was fully suppressed (Fig. 6c). This result indicates that cocktails of neutralising mAbs with non-overlapping epitopes can effectively suppress the selection of viral escape variants and therefore might be advantageous for clinical use. This conclusion is particularly evident considering that we observed viral escape not only in *in vitro* assays, but also in the *in vivo* setting. The mAb MB025_D01K is a non-neutralising mAb, however, it was the strongest Omicron RBD binder with K_D_ = 9.33 × 10^−10^ M and thus showed sub-nanomolar affinity (Fig. S2). We hypothesised that a non-neutralising mAb, when combined with a neutralising mAb in a cocktail, may enhance the activation of innate immune effector functions. In order to test this hypothesis, we first characterised the effector functions of the six analysed mAbs by using assays that measure antibody-dependent complement deposition (ADCD), cellular (THP-1) phagocytosis (ADCP), neutrophil phagocytosis (ADNP), and NK cell activation (ADNKA). While MB025_D01K was slightly weaker in promoting ADCD, ADCP, and ADNP than the other mAbs, it was a strong mediator of ADNKA (Fig. 6d). When MB025_D01K was formulated in a 1:1 mixture with MB021_C09K, this cocktail outperformed the single mAbs with respect to ADNP and ADNKA (Fig. 6e). This result indicates that by combining neutralising and non-neutralising mAbs that bind to the same antigen, stronger effector functions such as activation of neutrophils and NK cells are detected as if either of the mAbs was used individually.

## Discussion

Monoclonal antibodies are promising drug candidates for the prevention and therapy of various infectious diseases. During the course of the COVID-19 pandemic, substantial efforts were undertaken to develop mAbs as medicinal products that target the SARS-CoV-2 spike RBD and correspondingly neutralise the virus. However, the emergence of VOC posed an extraordinary challenge to the development of mAbs and many mAb-based products are no longer effective due to viral escape.^3^ Another risk that accompanies the treatment with mAbs is the possibility that the virus may mutate within the body and continues to infect the patient despite the presence of neutralising mAbs. This scenario was described in patients treated with Bamlanivimab in a randomised phase II clinical trial, where various mutations at position E484 in the spike protein emerged that lead to viral rebound and worsened symptoms.^21^ Here, we observed a similar scenario during the pre-clinical development of the mAb MB027_E04L in the hamster model. Therefore, we aimed to study the propensity of mAbs to select viral escape variants in a more comprehensive manner.

To this end, we investigated the effect of SHM in mAbs on the viral escape profile using two mAbs that we reverted back to their VH3-66 and VH3-53-based germline sequences. We found that germline mAbs had a generally lower affinity than matured mAbs. This is not surprising, because any affinity maturation that shaped mAb functions was effectively reverted by removing SHM. Nevertheless, some germline mAbs exerted surprisingly high affinities towards SARS-CoV-2.^22^ Our results are in accordance with studies that reverted large numbers of somatically hypermutated SARS-CoV-2 neutralising mAbs back to germline.^8^^,^^9^^,^^23^ Similarly, germline reverted mAbs were tested already against dengue virus, HIV, and HCV.^24^, ^25^, ^26^ Recently it has been described also by others that VOC preferentially escape from germline mAbs, while hypermutated mAbs still cross-neutralise certain VOC such as Omicron.^9^ Using highly quantitative SPR, we fine-characterised two such cases in more detail and found that in one case, MB025_A07K, SHM indeed caused an increase in affinity specifically against the Alpha variant, likely explaining why the mature mAb neutralised the Alpha variant, while the germline did not.^7^ In the second case, MB025_A09L, SHM increased the overall affinity of the mAb against the original antigen. This effect might cause cross-neutralisation of the Delta variant in an indirect manner, because the mAb effectively outcompetes the RBD-ACE2 interaction only when carrying SHM.^7^

Affinity maturation may not only enhance the affinity of an antibody against its antigen, but B cells can also mature in order to diversify their repertoire.^27^ We recently resolved an entire B-cell clonotype comprising 40 different mAbs, in which each mAb differed regarding the number and positions of SHM from the others. When testing against SARS-CoV-2 VOC, we found that some mAbs from the clonotype neutralised VOC, while others did not.^10^ In this study, we tested mAbs from this clonotype for their capacity to select viral escape and we found that clonotype diversification can change the amino acid positions at which viral escape occurs. This observation supports the concept that stochastic clonotype diversification is an evolutionarily advantageous trait. It results in a mixture of different antibodies, which are derived from one and the same common ancestor B cell, and that has the capacity to suppresses the stochastic formation of viral escape. While B-cell responses are already highly polyclonal in nature, this “anticipatory maturation” adds another level of diversity to the antibody repertoire.

The problem of rapid viral escape can be counteracted by combining at least two mAbs that select different profiles of viral escape into one cocktail, which decreases the statistical likelihood that a virus escapes from neutralisation by both mAbs. This concept was successfully exploited in the mAb cocktail REGN-COV2.^14^ Single polymorphisms that lead to viral escape from both mAbs in the REGN-COV2 cocktail are statistically very rare, but still may occur.^28^ We confirmed this concept by using a mAb cocktail made up of the mAbs MB021_C09K and MB025_A09L. Furthermore, our epitope binning analysis revealed that a third mAb, MB027_D01K, could simultaneously bind the antigen together with two neutralising mAbs. We demonstrated that such non-neutralising mAb could synergistically work together with a neutralising counterpart to potentiate Fc-mediated effector functions such as neutrophil and NK cell activation. It is increasingly appreciated that Fc-mediated effector functions of antibodies are an important feature of vaccine-induced immunity towards SARS-CoV-2,^29^ and also mAbs may benefit from an intact and functional Fc domain.^30^ Particularly in the context of therapeutic purposes, i.e., administration of a mAb into an already infected individual, the Fc-mediated effector functions play a vital role.^31^ The total number of antibodies that can be formulated into a cocktail of simultaneously binding mAbs depends on the size of the antigen and for SARS-CoV-2, cocktails with up to three mAbs have already been reported.^32^^,^^33^

A more thorough understanding of the specific SHM that lead to an enhanced binding of a certain virus variant may pave the way into an era of mAb products that can be adapted to upcoming virus variants. We recently associated two amino acid substitutions in the CDR1 and another one in the CDR3 of the B-cell clonotype tested here with a gain-of-neutralisation phenotype against the Beta and Gamma variants of SARS-CoV-2.^10^ The feasibility of the concept was further demonstrated by adapting the mAb Tixagevimab to the Omicron variant using a single amino acid substitution.^9^

### Limitations

The effect of SHM on mAb binding quality can be examined by reversion of point mutations that are identified by alignment with the germline database. This process reliably works only for the variable heavy (V_H_) and light (V_L_) genes, because the CDR3 at the V(D)J junction site contains non-template mutations for which the original sequence in the naïve ancestor B cell cannot be reconstructed. This limits our conclusion on SHM in the V_H_ and V_L_ genes and additional effects might play a role for SHM that potentially exist in the CDR3. The germline conversion was successfully used in a similar manner by others.^9^ Furthermore, we can rule out the discussed limitations for our comparison of MB027_D06L and CL39, because both sequences were derived from authentic B cells found in the same blood donor and each mAb carries unique SHM also in the CDR3.

We suspected that the SPR assay we performed here might be prone to exaggerate the mAb affinity due to the presence of multimers and aggregates. This problem was addressed by a second purification step via SEC to specifically isolate the dimeric IgG1 mAb species. Nevertheless, it cannot be fully excluded that the mAbs slowly started to aggregate again after SEC. To allow the maximal comparability between our samples, we chose to immobilise the antigens in the SPR analysis and use the mAbs as analyte. In such a scenario, avidity effects cannot be ruled out, however, we immobilised the antigen at a low density, so that avidity effects are not expected to occur.

When analysing *in vitro* viral escape mutants, we often detected multiple mutants appearing simultaneously in the samples. In such cases, Sanger sequencing of the amplicons is not able to discriminate between mutations that occur in separate viruses and mutations that co-occur in the same viral genome. To resolve for the formation of quasi-species during the experiment, we sequenced the same samples by Oxford Nanopore long-read sequencing. Nevertheless, the generated amount of data was not sufficient to provide clear hints for the presence of epistatic effects between different escape mutations, i.e., that the escape can only occur when two mutations simultaneously appear together to maximise the trade-off between escape from the mAb and viral fitness. However, such epistatic interactions can be deducted from high-throughput surveillance of sequencing data.^34^ We studied the *in vitro* viral escape in two independent replicates of the same experiment. Although for the same mAbs we detected many similar viral escape variants, there were also cases where viral escape from a certain mAb was achieved by different mutations in the two experiments. These observations illustrate that viral escape is a stochastic process with massive biological variation and therefore cannot be fully recapitulated in independent experiments.

Our conclusion that combination of neutralising and non-neutralising mAbs could have synergistic effects *in vivo* is currently based only on data that was generated *in vitro*. The Fc-function of human IgG1 mAbs improves the outcome of hamster challenge experiments,^31^ but additional studies are needed to investigate the potential effect of non-neutralising mAbs on viral escape *in vivo*.

## Contributors

Conceptualisation: M.B., M.O., U.K.

Methodology: M.B., M.O., A.D.M.E.O., A.Sa. M.G.H., M.M., Y.B., AE.M., G.Z.

Investigation: M.B., M.O., A.D.M.E.O., A.Sa., W.R., W.B., M.G.H., M.M., I.H.

Visualisation: M.B., A.Sa., W.R.

Funding acquisition: M.B., A.Sc., A.D.M.E.O., U.K.

Project administration: M.B., U.K.

Supervision: U.K.

Writing – original draft: M.B.

Writing – review & editing: all authors.

All authors read and approved the final version of the manuscript. The underlying primary data were accessed and verified by M.B., A.Sa., and U.K.

## Data sharing statement

The raw data of the Oxford Nanopore sequencing experiment are openly available in fastq format effective immediately under https://doi.org/10.5281/zenodo.15269242. The code that was used for analysis of these sequencing data is provided under https://github.com/memumab/escapster/ and https://doi.org/10.5281/zenodo.15281608. Additional information is available upon reasonable request.

## Declaration of interests

The authors declare no conflict of interest.

## Appendix A Supplementary data

Figs. S1 and S2

Reagent validation file for cell lines and antibodies
