# Supplementary material for: Somatic hypermutation shapes the viral escape profile of SARS-CoV-2 neutralising antibodies
Source: eBioMedicine. 2025 May 21;116:105770. doi: 10.1016/j.ebiom.2025.105770 (PMC12148588; doi:10.1016/j.ebiom.2025.105770)
Supplement: Figs. S1 and S2 [file mmc1.docx]

**SUPPLEMENTARY MATERIAL**

**Supplementary Figures**

**
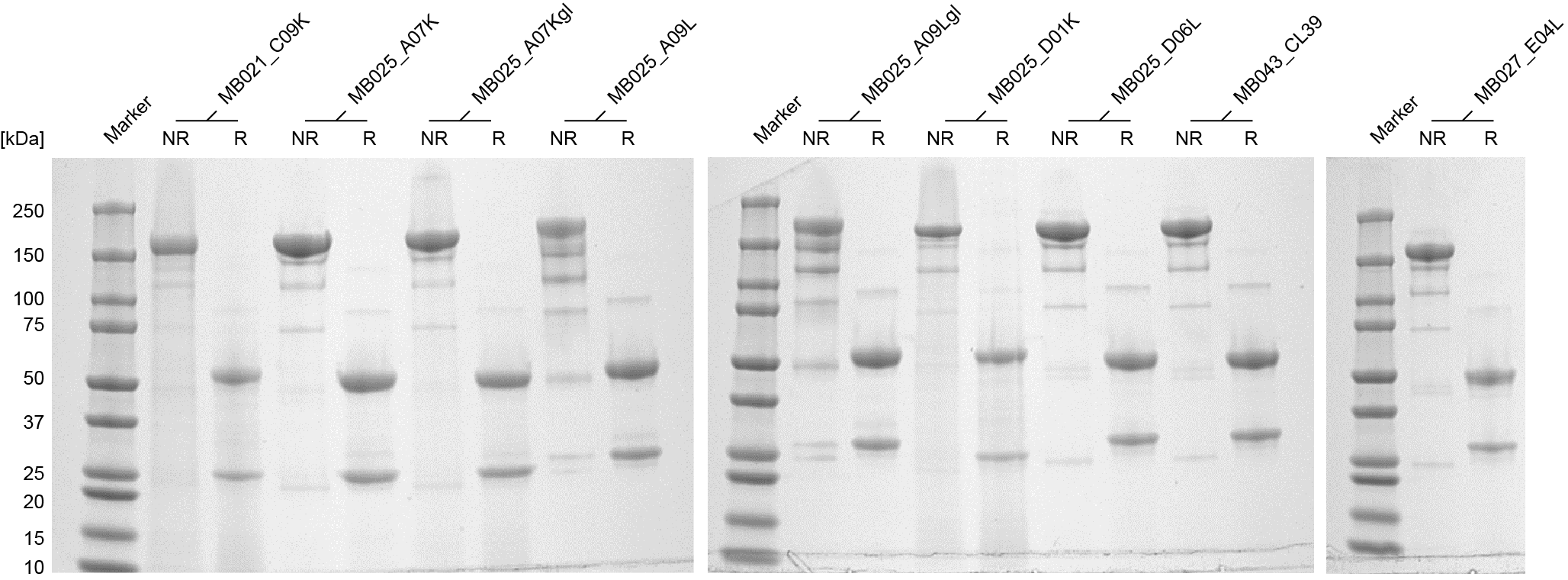
**

**Fig. S1. Gel electrophoresis of mAbs after affinity chromatography.** 10 µg of the protein eluates were loaded into each lane of the SDS-Page acrylamide gels. Each mAb was assessed under non-reducing (NR) and reducing (R) conditions. The gels were stained with Coomassie blue to visualize the protein bands.

**
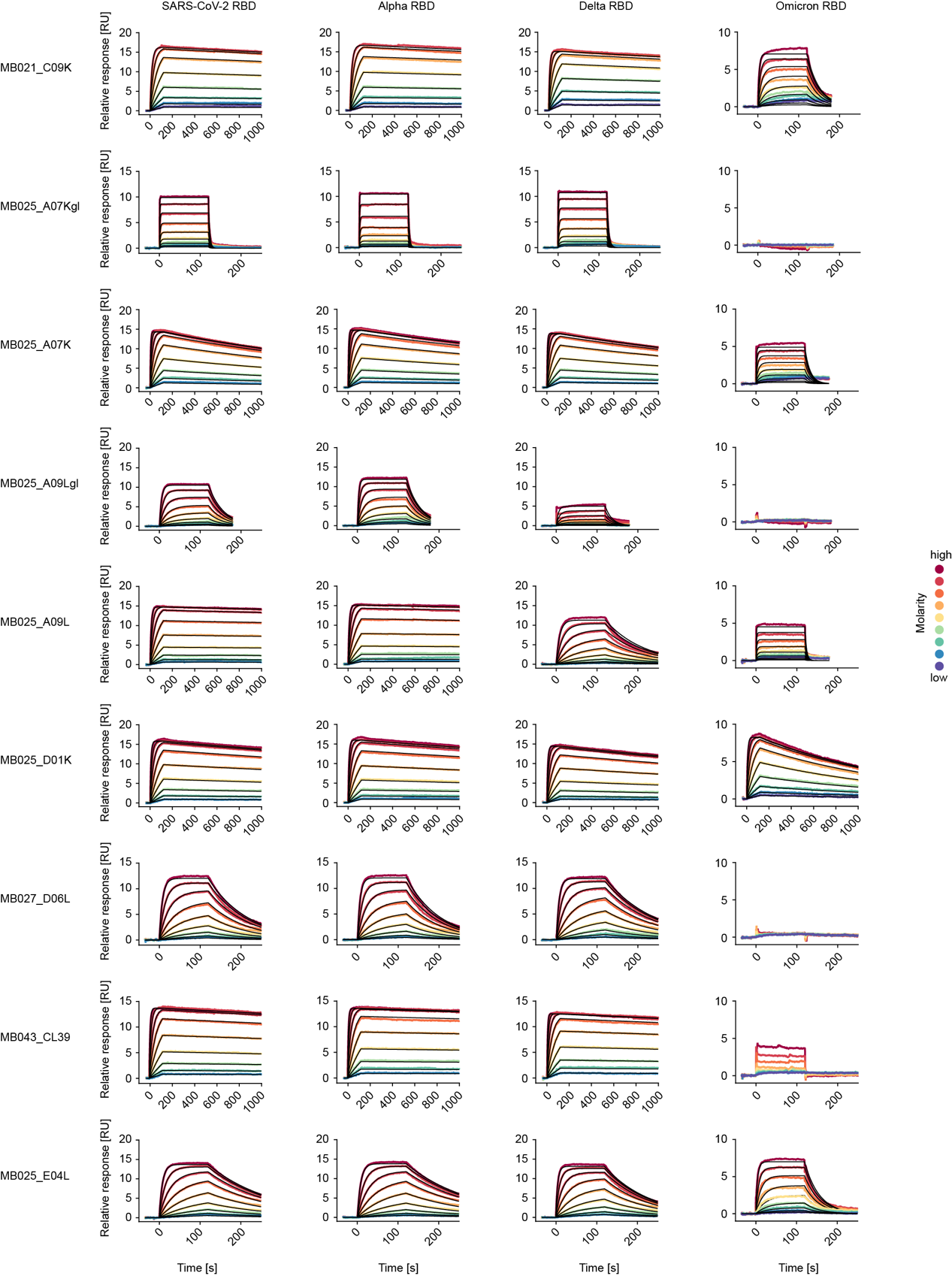
**

**Fig. S2. Complete set of quantitative SPR measurements carried out for this study.** Quantitative affinity determination of nine mAbs using SPR. The four columns represent four antigen variants to be immobilized on the chip.
