# Supplementary material for: Somatic hypermutation shapes the viral escape profile of SARS-CoV-2 neutralising antibodies
Source: eBioMedicine. 2025 May 21;116:105770. doi: 10.1016/j.ebiom.2025.105770 (PMC12148588; doi:10.1016/j.ebiom.2025.105770)
Supplement: Reagent validation file for cell lines and antibodies [file mmc2.pdf]

19.03.25
Cell line and antibody validation file for EBIOM-D-24-04262

Cell lines	Vendor	Cat. No.	RRID	Lot	Certificate	Mycoplasm test
ExpiCHO	ThermoFisher	A29127	CVCL_5J31	2332043	attached	negative
Calu-3	ATCC	HTB-55	CVCL_0609	70035668	attached	negative
Vero-B4	DSMZ	ACC 33	CVCL_1912	13	attached	negative
THP-1	DSMZ	ACC 16	CVCL_0006	38	attached	negative

Flow cytometry antibodies	Vendor	Cat. No.	RRID
FITC IgG Goat Anti-Guinea Pig Complement C3	MP Bio	855385	AB_2334913
anti-human CD66b-Pacific Blue antibody	Biolegend	305111	AB_2563294
BV605 anti-human CD107a	Biolegend	328634	AB_2563851
anti-human CD3-APC-Cy7	Biolegend	300426	AB_830755
anti-human CD56-PE-Cy7	BD Bioscience	335826	AB_2857328
anti-human MIP-1β-BV421	BD Bioscience	562900	AB_2737877
anti-human IFN-γ-PE	Biolegend	506507	AB_315440

Antibodies produced in this study	Expression system	Plasmid sequence verified	Purity confirmed	Binding validated
MB025_A07K	ExpiCHO	yes	SDS-Page/Coomassie/SEC	ELISA, SPR
MB025_A09L	ExpiCHO	yes	SDS-Page/Coomassie/SEC	ELISA, SPR
MB025_E04L	ExpiCHO	yes	SDS-Page/Coomassie/SEC	ELISA, SPR
MB021_C09K	ExpiCHO	yes	SDS-Page/Coomassie/SEC	ELISA, SPR
MB027_D06L	ExpiCHO	yes	SDS-Page/Coomassie/SEC	ELISA, SPR
MB025_D01K	ExpiCHO	yes	SDS-Page/Coomassie/SEC	ELISA, SPR
MB025_A07Kgl	ExpiCHO	yes	SDS-Page/Coomassie/SEC	ELISA, SPR
MB025_A09Lgl	ExpiCHO	yes	SDS-Page/Coomassie/SEC	ELISA, SPR
CL00	ExpiCHO	yes	SDS-Page/Coomassie	ELISA
CL01	ExpiCHO	yes	SDS-Page/Coomassie	ELISA
CL02	ExpiCHO	yes	SDS-Page/Coomassie	ELISA
CL03	ExpiCHO	yes	SDS-Page/Coomassie	ELISA
CL04	ExpiCHO	yes	SDS-Page/Coomassie	ELISA
CL05	ExpiCHO	yes	SDS-Page/Coomassie	ELISA
CL06	ExpiCHO	yes	SDS-Page/Coomassie	ELISA
CL07	ExpiCHO	yes	SDS-Page/Coomassie	ELISA
CL08	ExpiCHO	yes	SDS-Page/Coomassie	ELISA
CL09	ExpiCHO	yes	SDS-Page/Coomassie	ELISA
CL10	ExpiCHO	yes	SDS-Page/Coomassie	ELISA
CL11	ExpiCHO	yes	SDS-Page/Coomassie	ELISA
CL13	ExpiCHO	yes	SDS-Page/Coomassie	ELISA
CL14	ExpiCHO	yes	SDS-Page/Coomassie	ELISA
CL15	ExpiCHO	yes	SDS-Page/Coomassie	ELISA
CL16	ExpiCHO	yes	SDS-Page/Coomassie	ELISA
CL17	ExpiCHO	yes	SDS-Page/Coomassie	ELISA
CL18	ExpiCHO	yes	SDS-Page/Coomassie	ELISA
CL19	ExpiCHO	yes	SDS-Page/Coomassie	ELISA
CL20	ExpiCHO	yes	SDS-Page/Coomassie	ELISA
CL21	ExpiCHO	yes	SDS-Page/Coomassie	ELISA
CL22	ExpiCHO	yes	SDS-Page/Coomassie	ELISA
CL23	ExpiCHO	yes	SDS-Page/Coomassie	ELISA
CL24	ExpiCHO	yes	SDS-Page/Coomassie	ELISA
CL25	ExpiCHO	yes	SDS-Page/Coomassie	ELISA
CL26	ExpiCHO	yes	SDS-Page/Coomassie	ELISA
CL27	ExpiCHO	yes	SDS-Page/Coomassie	ELISA
CL28	ExpiCHO	yes	SDS-Page/Coomassie	ELISA
CL29	ExpiCHO	yes	SDS-Page/Coomassie	ELISA
CL30	ExpiCHO	yes	SDS-Page/Coomassie	ELISA
CL31	ExpiCHO	yes	SDS-Page/Coomassie	ELISA
CL32	ExpiCHO	yes	SDS-Page/Coomassie	ELISA
CL33	ExpiCHO	yes	SDS-Page/Coomassie	ELISA
CL34	ExpiCHO	yes	SDS-Page/Coomassie	ELISA
CL35	ExpiCHO	yes	SDS-Page/Coomassie	ELISA
CL36	ExpiCHO	yes	SDS-Page/Coomassie	ELISA
CL37	ExpiCHO	yes	SDS-Page/Coomassie	ELISA
CL38	ExpiCHO	yes	SDS-Page/Coomassie	ELISA
CL39	ExpiCHO	yes	SDS-Page/Coomassie/SEC	ELISA, SPR

Thermo Fisher SCIENTIFIC

Certificate of Analysis

ExpiCHO™-S Cells

Product No. A29127
Lot No. 2332043
Date of Manufacture 04Mar2021

Viability

Specification: Cells must be recovered as healthy logarithmically growing cells within 3 to 4 days post thawing. Viability is measured and must be $\geq$90%.

Result: Meets specification

Mycoplasma Testing

Specification: Test results must indicate the absence of mycoplasma.

Result: Meets specification

Sterility Testing

Specification: Negative

Result: Meets Specification

For Research Use Only. Not for use in diagnostic procedures. If you have any further questions about this Certificate of Analysis, please contact Technical Services at 1-800-955-6288 (US and Canada) or 1-760-603-7200, x2 (all other countries).

Thermo Fisher Scientific
Life Sciences Solutions
5781 Van Allen Way
Carlsbad, CA, USA 92008
www.thermofisher.com
For inquiries, contact us at cofarequests@thermofisher.com

Shannon Orr
Sr. Manager, Quality
Issued on 18 May 2021

CERTIFICATE OF ANALYSIS

ATCC® Number: **HTB-55™**

Lot Number: **70035668**

Name: **Calu-3**

Description: **Lung Adenocarcinoma**

Species: **Human (*Homo sapiens*)**

Volume/Ampule: **Approximately 1 mL**

Date Frozen: **11JUN2020**

Recovery: **A T-75 setup at a seeding density of 3.0 x 10^4 viable cells/cm^2 reaches approximately 10% confluence in 1 day and 80% confluence in 9 days.**

Product Format: **Cells cryopreserved in the appropriate cryopreservation medium**

Expiration Date: **Not applicable**

Storage Conditions: **Vapor phase of liquid nitrogen**

Test / Method	Specification	Result
**Ampule passage number**	Report results	17
**Population doubling level (PDL)**	Report results	Not applicable
**Total cells/ampule**<br>(Cell count using Trypan Blue stain method)	Report results	3.4 x 10^6 total cells/ampule
**Post-freeze viability**<br>(Cell count using Trypan Blue stain method)	≥ 70.0%	90.1%
**Growth properties**<br>(Visual observation method)	Adherent	Adherent
**Morphology**<br>(Visual observation method)	Epithelial-like*	Epithelial-like
**Test for mycoplasma contamination**<br>Hoechst DNA stain (indirect) method<br>Agar culture (direct) method<br>PCR-based assay	 None detected<br>None detected<br>None detected	 None detected<br>None detected<br>None detected
**Species determination: COI assay (interspecies)**	Human	Human

ATCC
10801 University Boulevard
Manassas, VA 20110-2209 USA
www.atcc.org

800-638-6597 or 703-365-2700
Fax: 703-365-2750
E-mail: tech@atcc.org
or contact your local distributor

CERTIFICATE OF ANALYSIS

ATCC® Number: **HTB-55™**

Lot Number: **70035668**

**Species determination: STR analysis (intraspecies)**	**Human (Unique DNA Profile)**<br>TH01: 6, 9.3<br>D5S818: 11<br>D13S317: 12<br>D7S820: 10, 11<br>D16S539: 12, 14<br>CSF1PO: 11, 12<br>Amelogenin: X<br>vWA: 16, 17<br>TPOX: 8	**Human (Unique DNA Profile)**<br>TH01: 6, 9.3<br>D5S818: 11<br>D13S317: 12<br>D7S820: 10, 11<br>D16S539: 12, 14<br>CSF1PO: 11, 12<br>Amelogenin: X<br>vWA: 16, 17<br>TPOX: 8
**Sterility test (BacT/ALERT 3D)**<br>iAST bottle (aerobic) at 32.5°C<br>iNST bottle (anaerobic) at 32.5°C	No growth<br>No growth	No growth<br>No growth
**Human pathogenic virus testing**<br>(PCR-based assay for HIV, HepB, HPV, EBV, and CMV)	Report results	HIV – None detected<br>HepB – None detected<br>HPV – None detected<br>EBV – None detected<br>CMV – None detected

* Epithelial-like: Any adherent cells of a polygonal shape with clear, sharp boundaries between them.

Product meets European Union requirements for production of technical blood products.

Revised on 20Aug2020 to correct error in the recovery statement.

Quality Assurance Specialist; Quality Assurance

ATCC hereby represents and warrants that the material provided under this certificate is pure and has been subjected to the tests and procedures specified and that the results described, along with any other data provided in this certificate, are true and correct to the best of the company's knowledge and belief. This certificate does not extend to the growth and/or passage of any living organism or cell line beyond what is supplied within the container received from ATCC.

This product is intended to be used for laboratory research use only. It is not intended for use in humans, animals, or for diagnostics. Appropriate Biosafety Level (BSL) practices should always be used with this material. Refer to the Product Information Sheet for instructions on the correct use of this product.

ATCC products may not be resold, modified for resale, used to provide commercial services, or to manufacture commercial products without prior written agreement from ATCC.

© 2017 ATCC. The ATCC trademark and trade name are owned by the American Type Culture Collection.

ATCC
10801 University Boulevard
Manassas, VA 20110-2209 USA
www.atcc.org

800-638-6597 or 703-365-2700
Fax: 703-365-2750
E-mail: tech@atcc.org
or contact your local distributor

COI Species Report

Cell line: VERO-B4

ACC-No.: 033

Date of analysis: 13.10.2014

DNA processing number:

Methode: DNA Barcoding by PCR amplification of 5Â´coding region of cytochrome c oxidase I (658 bp fragment size). Cycle sequencing of respective PCR products revealed following assignment upon submission to BOLD (Ratnasingham, S., Hebert, P. D. N. (2007) BOLD: The Barcode of Life Data System (www.Barcodinglife.org). Molecular Ecology Notes, 2007; 7(3): 355-364

Primer:

1x LepF1_t1: ATT TAG GTG ACA CTA TAG ATT CAA CCA ATC ATA AAG ATA TTG G

1x VF1_t1: ATT TAG GTG ACA CTA TAG TCT CAA CCA ACC ACA AAG ACA TTG G

1x VF1d_t1: ATT TAG GTG ACA CTA TAG TCT CAA CCA ACC ACA ARG AYA TYG G

3x VF1i_t1: ATT TAG GTG ACA CTA TAG TCT CAA CCA ACC ANA ANG ANA TNG G

1x LepR1_t1: TAA TAC GAC TCA CTA TAG GGT AAA CTT CTG GAT GTC CAA AAA ATC A

1x VR1d_t1: TAA TAC GAC TCA CTA TAG GGT AGA CTT CTG GGT GGC CRA ARA AYC A

1x VR1_t1: TAA TAC GAC TCA CTA TAG GGT AGA CTT CTG GGT GGC CAA AGA ATC A

3x VR1i_t1: TAA TAC GAC TCA CTA TAG GGT AGA CTT CTG GGT GNC CNA ANA ANC A

Sequence:

5`-AAGMTTGGACTCTATACCTACTATTCGGTGCATGAGCTGGAAYCATAGGAACAGCTCTAA

GCCTTCTCATTCGAGCTGAATTAGGCCAACCCGGTAGTTTACTAGGCAGTGACCATATCT

ATAATGTCATTGTAACAGCCCATGCATTTATTATAATTTTCTTCATAGTTATACCCATTA

TAATCGGAGGGTTCGGGAACTGACTAGTACCCTTGATAATTGGTGCTCCTGACATAGCAT

TTCCCCGTCTAAATAATATGAGCTTCTGACTTCTTCCCCCCTCCTTCCTGCTGCTAATGG

CATCAACCATAATCGAGGCTGGCGCTGGAACAGGTTGAACAGTATACCCCCCCTTAGCAG

© DSMZ - All rights reserved

GAAACCTCTCTCACCCAGGGGCCTCCGTAGACTTAGTTATTTTCTCCCTCCACCTAGCAG

GAGTTTCCTCTATCCTGGGGGCTATCAACTTCATTACCACCATTATCAACATGAAGCCCC

CCGCCATATCCCAGTATCAAACCCCGTTATTTGTCTGATCTGTCCTAATCACAGCAATCC

TACTACTCCTCTCCCTGCCAGTCTTAGCTGCCGGCATTACTATACTATTAACAGACCGCA

ACCTCAACACTACCTTCTTTGATCCTACTGGAGGGGGAGACCCTATCCTATACCAACACC

TATTTTGATTYTTYGGMCAYCCAGAAGTYTACCCTATAGTGAGCGKWWWWAAA-3`

Taxonomic Level Taxon Assignment Probability of Placement (%)
Phylum Chordata 100
Class Mammalia 100
Order Primates 100
Family Cercopithecidae 100
Genus Chlorocebus 100

Identification Summary:

Search Result: A species level match could not be made, the queried specimen is likely to be one of the following:

Chlorocebus sabaeus

Cercopithecus aethiops

© DSMZ - All rights reserved

Leibniz-Institut
DSMZ - Deutsche Sammlung von
Mikroorganismen und Zellkulturen GmbH

VERO-B4

Certificate of Origin and Analysis

**Cell line model**	kidney
**Catalogue number**	DSMZ ACC 33
**Preferential origin**	The exporter of the products covered by this document declares that the goods, except where otherwise clearly indicated, are of German preferential origin.
**Origin**	established from the kidney of a normal adult African green monkey in Japan in 1962; cells are susceptible to a number of viruses; used in virus replication studies and plaque assays, and as indicator cell line for mycoplasma testing
**Biosafety level**	1
**Viral status**	ELISA: reverse transcriptase negative; PCR: EBV -, HBV -, HCV -, HIV-1 -, HIV-2 -, HTLV-1/2 -, SMRV -
**Medium**	90% RPMI 1640 + 10% h.i. FBS

Testing specification for lot number 13 / date: 06.09.2021

**Sterility**	14 d culture free of antibiotics	passed	15.11.2021
**Mycoplasma**	PCR assays including internal and negative controls	passed	21.09.2021
**Species specificity**	COI DNA Barcoding	authentic	

Short tandem repeat profile

For most animal cell lines there is unfortunately no STR typing system available for individualization within a species.

Species identification:

The correctness of the species origin was checked using the Cytochrome C Oxidase subunit I (COI) method:

Chlorocebus aethiops (Cercopithecus aethiops)

Identity analysis reviewed by:

Dr. W. Dirks

Mycoplasma detection reviewed by:

Dr. U. Rand

Geschäftsführung/Directors:
Prof. Dr. Jörg Overmann
Bettina Fischer
Aufsichtsratsvorsitzender/Head of
Supervisory Board: MR Dr. David Schnieders

Braunschweigische Landessparkasse
(NORD/LB) Kto.-Nr./Account: 2 039 220
BLZ/Bank Code: 250 500 00
IBAN DE22 2505 0000 0002 0392 20
SWIFT (BIC) NOLADE 2 H

Handelsregister/
Commercial Register:
Amtsgericht Braunschweig
HRB 2570
Steuer-Nr. 13/200/24030

Leibniz-Institut
DSMZ - Deutsche Sammlung von
Mikroorganismen und Zellkulturen GmbH

THP-1

Certificate of Origin and Analysis

Cell line model acute monocytic leukemia

Catalogue number DSMZ ACC 16

Preferential origin The exporter of the products covered by this document declares that the goods, except where otherwise clearly indicated, are of German preferential origin.

Origin established from the peripheral blood of a 1-year-old boy with acute monocytic leukemia (AML) at relapse in 1978; the cells can be used for induction of differentiation studies; the cells were described in the literature to produce lysozyme and to be phagocytic; carries t(9;11)(p21;q23) leading to KMT2A-MLLT3 (MLL-MLLT3; MLL-AF9) fusion gene. Exome and RNA sequence data are available (see Ref 18187 and Exome sequence

Biosafety level 1

Viral status ELISA: reverse transcriptase negative; PCR: EBV -, HBV -, HCV -, HHV-8 -, HIV-1 -, HIV-2 -, HTLV-1/2 -, MLV -, SMRV -

Medium 80-90% RPMI 1640 + 10-20% h.i. FBS

Testing specification for lot number 38 / date: 16.09.2022

**Sterility**	14 d culture free of antibiotics	passed	17.11.2022
**Mycoplasma**	PCR assays including internal and negative controls	passed	27.09.2022
**Identity**	short tandem repeat microsatellite genotyping - STR17	authentic	
**MSI Status**	Microsatellite Instability (MSI) testing	negative	

Short tandem repeat profile

**STR Loci**	D5S818	D13S317	D7S820	D16S539	vWA	TH01	TPOX	CSF1	D3S1358
**Allels**	11 12	8 13	10 10	11 12	16 17	8 9.3	8 11	11 13	15 17

**STR Loci**	D21S11	D18S51	Penta D	Penta E	D8S1179	FGA	D19S433	D2S1338	Amelogenin
**Allels**	30 31.2	13 14	10 12	11 15	10 14	24 25	13 13	17 18	X Y

Identity analysis reviewed by:

Dr. W. Dirks

Mycoplasma detection reviewed by:

Dr. U.Rand

Geschäftsführung/Directors:
Prof. Dr. Jörg Overmann
Bettina Fischer
Aufsichtsratsvorsitzender/Head of
Supervisory Board: MR Dr. David Schnieders

Braunschweigische Landessparkasse
(NORD/LB) Kto.-Nr./Account: 2 039 220
BLZ/Bank Code: 250 500 00
IBAN DE22 2505 0000 0002 0392 20
SWIFT (BIC) NOLADE 2 H

Handelsregister/
Commercial Register:
Amtsgericht Braunschweig
HRB 2570
Steuer-Nr. 13/200/24030
